# Deep learning improves image quality in motion-robust and sedation-free pediatric brain MRI

**DOI:** 10.1007/s00330-026-12482-y

**Published:** 2026-04-02

**Authors:** Anna Magdalena Baz, Zeynep Bendella, Christoph Katemann, Alois M. Sprinkart, Kilian Weiss, Oliver M. Weber, Johannes M. Peeters, Nils C. Lehnen, Ralf Clauberg, Julian A. Luetkens, Alexander Radbruch, Barbara Daria Wichtmann

**Affiliations:** 1 Department of Diagnostic and Interventional Neuroradiology, University Hospital Bonn, Bonn, Germany; 2 German Center for Neurodegenerative Diseases (DZNE), Bonn, Germany; 3 Philips GmbH Market DACH, Hamburg, Germany; 4 Department of Diagnostic and Interventional Radiology, University Hospital Bonn, Bonn, Germany; 5 Philips Healthcare, Best, The Netherlands

**Keywords:** Magnetic resonance imaging, Brain, Pediatrics, Deep learning, Motion artifacts

## Abstract

**Objectives:**

Motion and limited compliance compromise diagnostic MR image quality, particularly in pediatric patients who frequently require sedation. Single-shot sequences offer a time-efficient alternative but suffer from reduced image quality. This study aimed to evaluate the diagnostic performance of a deep learning (DL) framework combining compressed sensing (CS) and convolutional neural networks (CNNs) to enhance T2-weighted single-shot MRI (T2-SSH_DL_) compared with conventional CS-based reconstruction (T2-SSH_conv_) and routinely acquired high-resolution T2-weighted sequences.

**Materials and methods:**

This prospective single-center study included 62 pediatric patients (mean age, 7.4 ± 4.9 years; 36 males, 26 females), who underwent T2-weighted single-shot brain MRI (29 sedated, 33 awake). Raw data were reconstructed using a DL-based pipeline and compared with conventional CS-based reconstructions. Quantitative metrics included apparent contrast-to-noise ratio (aCNR), apparent signal-to-noise ratio (aSNR), and edge rise distance (ERD). Two radiologists rated images for artifacts, sharpness, lesion conspicuity, and overall quality on a 5-point Likert scale.

**Results:**

T2-SSH_DL_-sequences showed significantly higher aCNR (29.9 ± 22.6 vs. 26.7 ± 16.5; *p* < 0.001), aSNR (41.6 ± 27.9 vs. 38.2 ± 20.8; *p* = 0.003), and improved sharpness (ERD 0.90 ± 0.35 mm vs. 1.35 ± 0.42 mm; *p* < 0.001). Qualitative assessments confirmed superior image quality, lesion conspicuity, and sharpness (*p* < 0.001). Compared with high-resolution T2-weighted sequences, T2-SSH_DL_-sequences showed fewer motion artifacts and comparable lesion conspicuity in non-sedated patients.

**Conclusion:**

DL-based reconstruction significantly enhances the diagnostic quality of T2-weighted single-shot brain MRI in pediatric patients, enabling clinically usable, ultrafast, motion-robust imaging with potential to reduce the need for sedation.

**Key Points:**

***Question*** *Can deep learning-based reconstruction elevate motion-robust single-shot T2-weighted pediatric brain MRI to diagnostic image quality levels, enabling reliable imaging without sedation?*

***Findings**** Both quantitative and qualitative evaluations confirmed significantly improved image quality of deep learning-enhanced single-shot T2-weighted brain MRI compared with conventional reconstruction*.

***Clinical relevance**** Deep learning-enhanced reconstruction improves image quality in ultrafast, motion-robust single-shot pediatric brain MRI, potentially reducing the need for sedation while preserving diagnostic accuracy. This approach may enhance patient safety and shorten examination time in routine neuroimaging*.

**Graphical Abstract:**

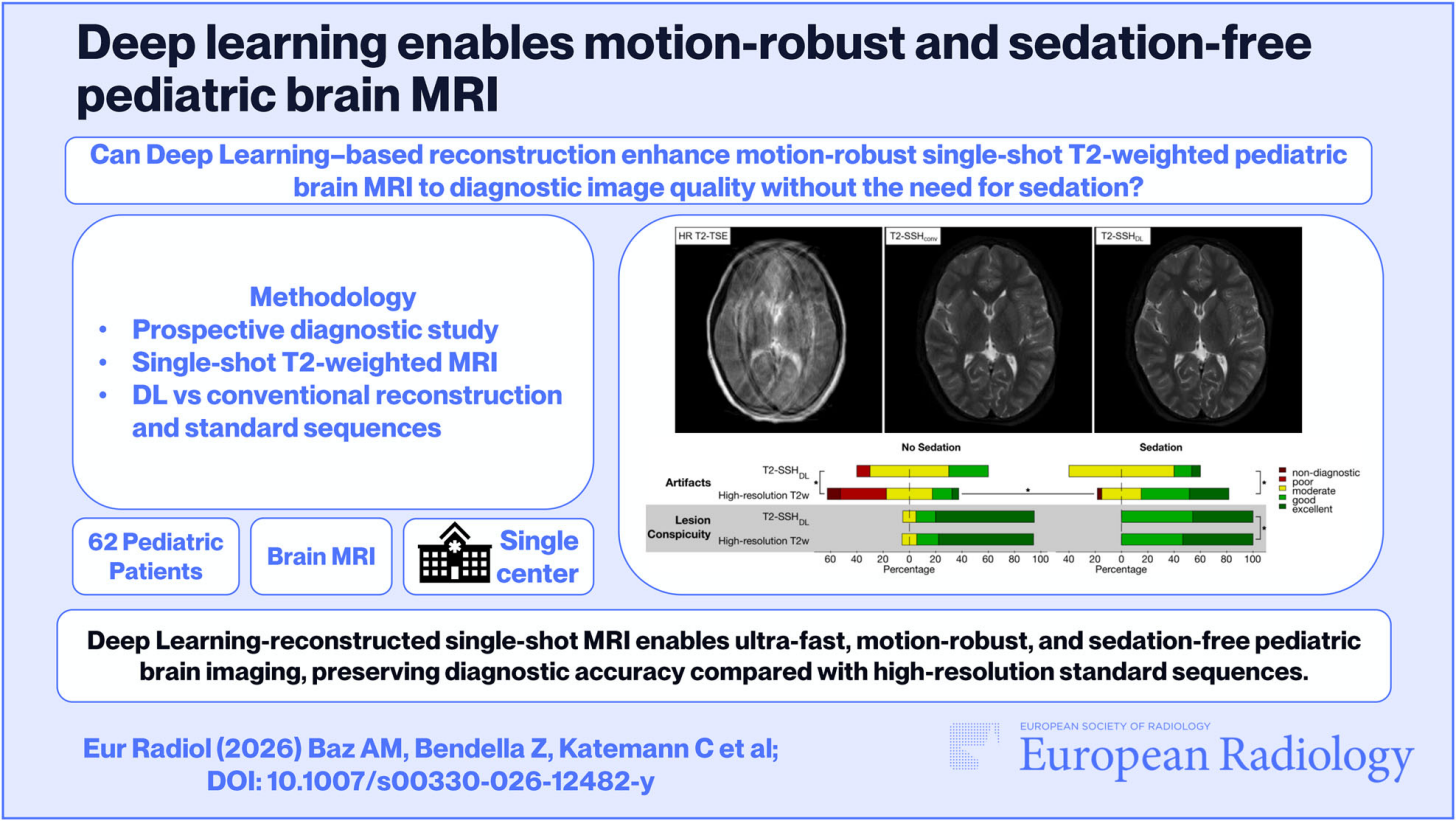

## Introduction

Fast, motion-robust MRI remains one of the major technological frontiers in diagnostic imaging, particularly in pediatric neuroimaging, where patient motion and limited compliance challenge the achievable acquisition speed and compromise image quality and often necessitate sedation [1–3]. Developing faster, motion-tolerant imaging techniques is therefore essential to improve safety, diagnostic quality, and workflow efficiency in pediatric radiology [4]. Although acceleration strategies such as parallel imaging, compressed sensing (CS) [5–7], and motion-compensated approaches like PROPELLER (periodically rotated overlapping parallel lines with enhanced reconstruction) imaging [8, 9] have increased acquisition speed and robustness, they remain limited by trade-offs in signal-to-noise ratio (SNR), spatial resolution, artifact robustness, and diagnostic reliability. For pediatric brain MRI, single-shot T2-weighted imaging enables rapid and motion-robust acquisition but suffers from long echo train length that reduces contrast resolution and fine structural detail [10, 11]. It therefore remains uncertain whether deep learning (DL)-based reconstruction can overcome these constraints to achieve both speed and diagnostic image quality, particularly in uncooperative or motion-prone children.

Several studies have already demonstrated substantial gains in sharpness, contrast-to-noise ratio, and artifact suppression across various anatomical applications, underscoring the broad technological relevance of DL-based reconstruction for diagnostic radiology [12, 13]. Ikebe et al demonstrated that ultrafast whole-brain T2-weighted imaging can be achieved in only 7 s using dual-type DL-based reconstruction with single-shot acquisition, highlighting the feasibility of combining rapid imaging with high diagnostic quality [14]. However, most studies have been conducted in adult populations under well-controlled conditions, and evidence in pediatric imaging remains limited [15]. This lack of pediatric data represents a critical gap, given the clinical importance of reducing sedation, minimizing motion artifacts, and maintaining diagnostic accuracy in children who require frequent neuroimaging for chronic or oncologic conditions. Addressing this gap is essential not only to establish the clinical utility and reliability of DL-based reconstruction in routine pediatric radiology but also to assess the physical and algorithmic limits of this emerging technology.

Building on these observations, we designed a prospective study to address this unmet need in pediatric neuroimaging. This study aimed to evaluate a novel DL-based reconstruction framework for T2-weighted single-shot brain MRI in a pediatric cohort that integrates CS acceleration with convolutional neural networks (CNNs) for denoising and resolution enhancement without increasing acquisition time. We hypothesized that this approach would improve image sharpness, contrast, and overall diagnostic quality compared with conventional CS-based reconstruction, thereby demonstrating the potential of DL-enhanced single-shot imaging to enable ultrafast, motion-robust, and sedation-free pediatric brain MRI [16, 17].

## Materials and methods

### Study population

This prospective study was approved by the institutional review committee of the University Hospital Bonn (ClinicalTrials.gov identifier NCT05820113) and included 62 pediatric patients (mean ± standard deviation (SD) age, 7.4 ± 4.9 years; age range, 0 to < 18 years, 36 males, 26 females) who underwent a clinically indicated brain MRI. Indications included epilepsy and movement disorders, neoplastic or vascular conditions, visual and auditory impairments, congenital disorders and anatomical abnormalities, increased intracranial pressure, former preterm infants, especially those with a history of intraventricular hemorrhage with or without the development of hydrocephalus, as well as other patients with hydrocephalus and MRI examinations to exclude intracranial pathology, e.g., in cases of unclear headache, suspected migraine, or as part of the evaluation prior to stem cell transplantation. Participants were recruited between October and December 2024. Written informed consent was obtained from all patients’ legal guardians prior to participation in the study. 29 patients were sedated (mean ± SD age, 3.1 ± 2.2 years, age range, 0–10 years) and 33 patients were awake during their MRI examination (mean ± SD age, 11.1 ± 3.3 years, age range, 5 to < 18 years).

### Data acquisition

All scans were performed on a 3.0-T scanner (Ingenia 3.0-T ElitionX, Philips Healthcare) using the 32-channel head coil (33 scans, 3 mm slice thickness) or the 16-channel head coil (29 scans, 4 mm slice thickness). Patient-specific MRI protocols were established to address the clinical questions. In addition, CS-accelerated T2-weighted single-shot sequences (T2-SSH_conv_-sequences) were acquired in the transverse (58 scans), coronal (53 scans), and sagittal (60 scans) planes for study purposes. The raw data of these sequences were reconstructed using a prototypical DL-based reconstruction framework provided by the vendor that combines and integrates CS with two different CNNs to generate denoised and upscaled images (T2-SSH_DL_-sequences). This algorithm (NGSA patch, SmartSpeed Precise, Philips Healthcare) was running on the scanner and includes the Adaptive-CS network for sparsity-constrained reconstruction with non-uniform random subsampling [18], and the SuperRes network to remove ringing artifacts and increase image resolution. For a more detailed description of the DL implementation, we refer to the supplementary material of Bisschoff et al [12]. Imaging parameters are listed in Table 1.

**Table 1:** Imaging parameters

Parameter	T2-SSH (*n* = 171)	T2 2 mm 1024 cs (*n* = 55)	T2 512 3 mm (*n* = 19)	T2 512 1.8 mm (*n* = 11)
Echo time (TE) (ms)	105	88	90	90
Repetition time (TR) (ms)	10,062	5842	3327	3887
Flip angle (degree, °)	90	90	90	90
Acquired pixel spacing (mm)	0.61 × 0.61	0.19 × 0.19	0.47 × 0.47	0.47 × 0.47
Reconstructed pixel spacing (mm)	0.48 × 0.48			
CS factor	2	2.2	1.8	1.8
Acquisition time (mean ± SD) (s)	20 ± 5	252 ± 77	166 ± 7	278 ± 60

Summary of imaging parameters for the T2-weighted sequences used in the study, including conventional CS-accelerated single-shot (T2-SSH_conv_) and deep learning-reconstructed single-shot (T2-SSH_DL_) acquisitions, as well as reference high-resolution T2-weighted scans. Parameters include echo time (TE), repetition time (TR), flip angle, pixel spacing, CS factor, and acquisition time. The smaller acquired pixel spacing in the T2 1024 sequence results from the reduced field of view (200 mm) compared with the T2 512 sequence (240 mm)

The DL-based reconstruction framework used in this study is a vendor-developed hybrid approach that combines compressed sensing with two sequential convolutional neural networks, as previously described in detail by Bischoff et al [12]. In brief, the reconstruction pipeline consists of an Adaptive-CS network for sparsity-constrained reconstruction from undersampled k-space data, followed by a dedicated super-resolution network designed to suppress ringing artifacts and enhance spatial resolution. A schematic illustration of the complete network architecture and reconstruction workflow is provided in Bischoff et al [12], including a graphical depiction of the individual processing steps.

### Quantitative image analysis

**Fig. 1:**
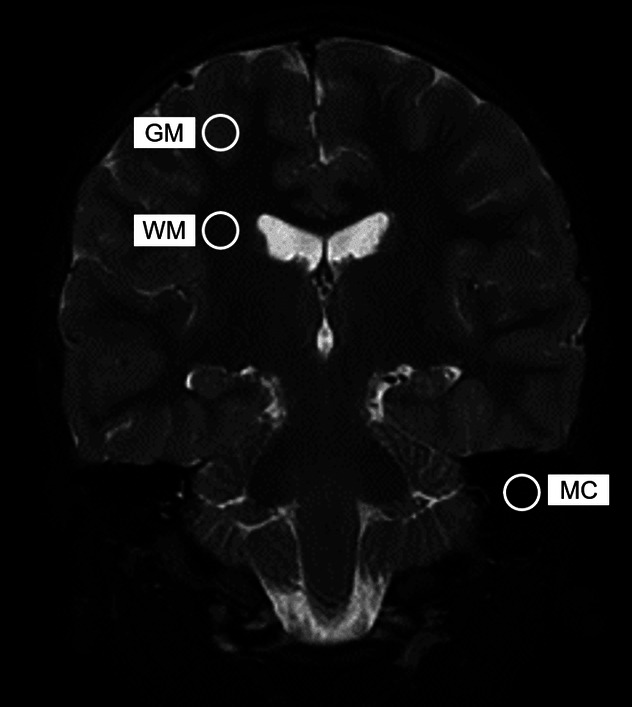
For calculating apparent contrast-to-noise ratio (aCNR) and apparent signal-to-noise ratio (aSNR), equal-sized regions of interest (ROIs) were consistently placed in the frontal cortex (GM), frontal white matter (WM), and mastoid cells (MC) at identical positions in both sequences in transverse, coronal and sagittal planes

**Fig. 2:**
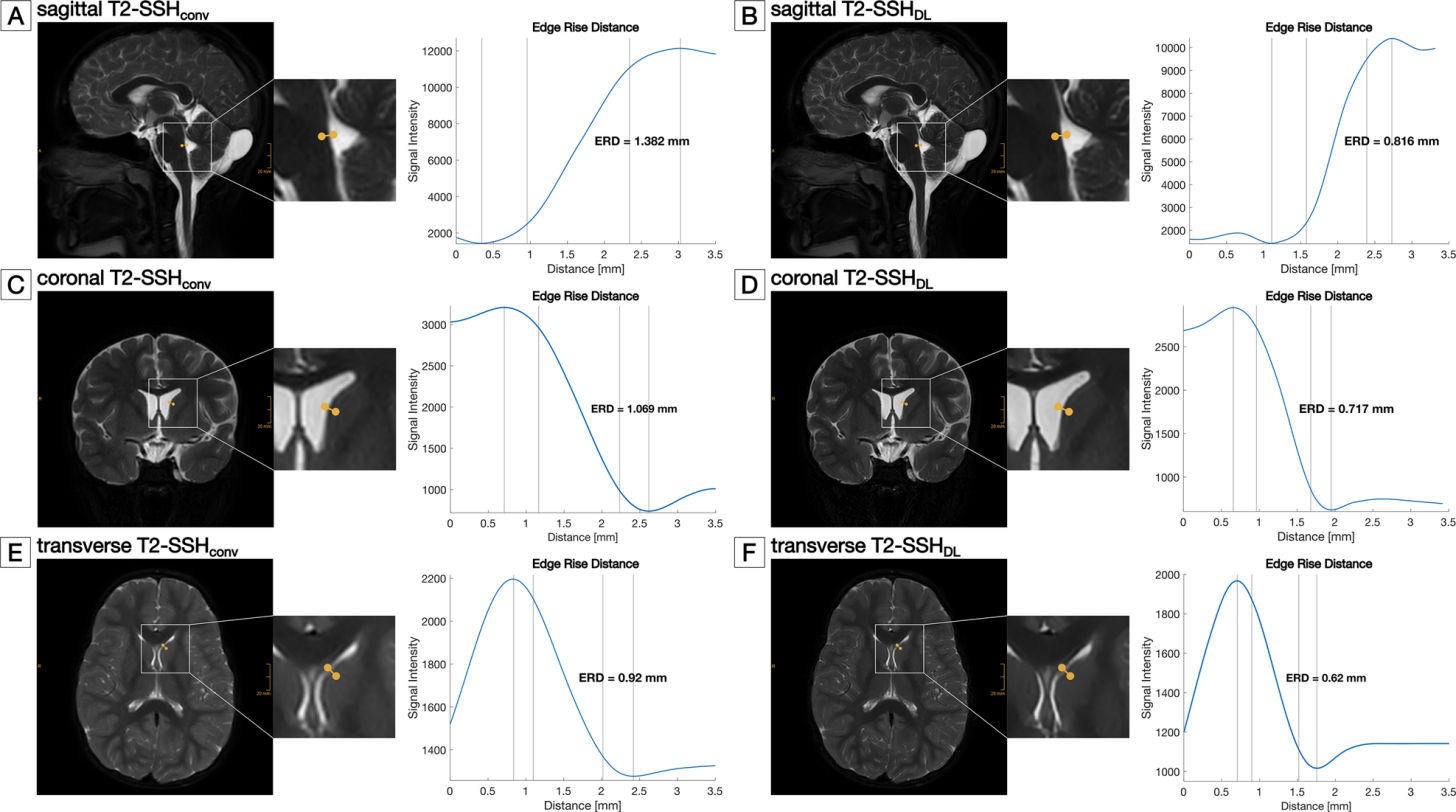
The edge rise distance (ERD) is derived from the signal intensity profile of a line drawn across the brainstem and the fourth ventricle in the sagittal planes (**A**, **B**) or alternatively across the anterior horn of the lateral ventricle and the head of the caudate nucleus in the coronal and transverse planes (**C**–**F**)

For quantitative analysis, equal-sized regions of interest (ROIs) were consistently placed in the frontal cortex (GM), frontal white matter (WM), and mastoid cells (MC) at identical locations in T2-SSH_conv_- and T2-SSH_DL_-sequences, as depicted in Fig. 1. Based on the image intensity distribution (mean ± SD) of these ROIs the apparent contrast-to-noise ratio (aCNR = (mean(GM) − mean(WM)) / SD(MC)) and apparent signal-to-noise ratio (aSNR = mean(WM) / SD(MC)) were calculated. In addition, the edge rise distance (ERD) was determined as a measure of image sharpness, derived from the signal intensity profile of a line drawn across the anterior horn of the lateral ventricle and the head of the caudate nucleus (in the transverse and coronal planes) or across the brainstem and the fourth ventricle (in the sagittal planes), as shown in Fig. 2. The ERD is calculated as the distance between the 10% and 90% signal intensity levels relative to the low and high signal intensity areas, represented by vertical lines in the graphs. A lower ERD indicates a sharper delineation of the abrupt signal change [12].

### Qualitative image analysis

For qualitative assessment, all images were analyzed by two radiologists with 4 (rater 1) and 9 (rater 2) years of experience, who independently rated T2-SSH_conv_- and T2-SSH_DL_-sequences qualitatively on a five-point Likert scale from 1 (non-diagnostic) to 5 (excellent) for the categories artifacts, image sharpness, lesion conspicuity, and overall image quality. In addition, the two radiologists compared T2-SSH_DL_-sequences with high-resolution T2-weighted sequences for the categories artifacts and lesion conspicuity, using the aforementioned five-point Likert scale. Five-point Likert items were defined with respect to the different categories as listed in Table 2. To assess intrarater reliability, both radiologists repeated the ratings for a second time at least 14 weeks after the initial rating.

**Table 2:** Definition of 5-point Likert scale per category

5-point Likert item	Artifacts	Image sharpness Lesion conspicuity Overall image quality
1	Non-diagnostic	Non-diagnostic, blurry
2	Severe artifacts with insufficient diagnostic confidence	Poor, structures can be defined but insufficient diagnostic confidence
3	Moderate artifacts, not interfering with diagnosis	Moderate, sufficient for diagnostic but low diagnostic confidence
4	Minimal artifacts	Good, diagnostic with high diagnostic confidence
5	No artifacts	Excellent, sharp images with exceptional diagnostic confidence

Description of the 5-point Likert-scale categories used for qualitative image assessment. Two radiologists independently rated both sequence types for artifacts, image sharpness, lesion conspicuity, and overall image quality

### Statistical analysis

Statistical analysis was performed in MATLAB (The MathWorks Inc. (2021), MATLAB Version: 9.11.0 (R2021b)) using paired-samples *t*-tests for paired continuous variables, including aCNR, aSNR, and ERD between T2-SSH_conv_- and T2-SSH_DL_-sequences.

Qualitative ratings on ordinal Likert scales were compared using the Wilcoxon signed-rank test for paired comparisons between T2-SSH_conv_- versus T2-SSH_DL_- and T2-SSH_DL_- versus high-resolution T2-weighted sequences. Between-group comparisons of independent samples for sedated versus non-sedated patients were performed using the Wilcoxon rank-sum test.

Interrater and intrarater reliability of qualitative image assessment was evaluated using Cohen’s Kappa with quadratic weighting, ranging from −1 to +1, where 0 indicates chance agreement, and 1 represents perfect agreement. According to Cohen, values ≤ 0 indicate no agreement, 0.01–0.20 slight, 0.21–0.40 fair, 0.41–0.60 moderate, 0.61–0.80 substantial, and 0.81–1.00 almost perfect agreement [19]. A *p*-value of < 0.05 was considered significant. Continuous variables of the quantitative assessment are provided as mean ± SD, and discrete variables of the qualitative assessment as median with interquartile range (IQR). Although median artifact scores with interquartile ranges may appear identical between sequences, statistical significance reflects paired, within-subject differences assessed by the Wilcoxon signed-rank test rather than differences in summary statistics alone.

## Results

### Quantitative evaluation

**Fig. 3:**
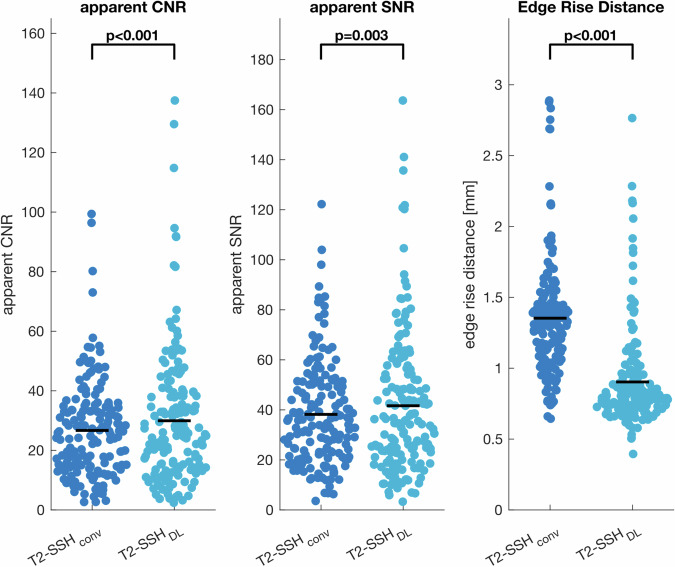
Comparison of quantitative parameters between conventional CS-reconstructed single-shot sequences (T2-SSH_conv_) and deep learning-enhanced single-shot sequences (T2-SSH_DL_). Bar plots show significantly higher apparent contrast-to-noise ratio (aCNR) and apparent signal-to-noise ratio (aSNR), and lower edge rise distance (ERD) for T2-SSH_DL_ (*p* < 0.05), indicating improved contrast, noise suppression, and sharpness

The mean aCNR and aSNR of T2-SSH_DL_-sequences were significantly higher than those of T2-SSH_conv_-sequences (aCNR 29.9 ± 22.6 vs. 26.7 ± 16.5; *p* < 0.001 and aSNR 41.6 ± 27.9 vs. 38.2 ± 20.8; *p* = 0.003). T2-SSH_DL_-sequences showed improved image sharpness compared with T2-SSH_conv_-sequences (ERD 0.90 ± 0.35 mm vs. 1.35 ± 0.42 mm, *p* < 0.001). Figure 3 summarizes all quantitative results.

### Qualitative evaluation

**Fig. 4:**
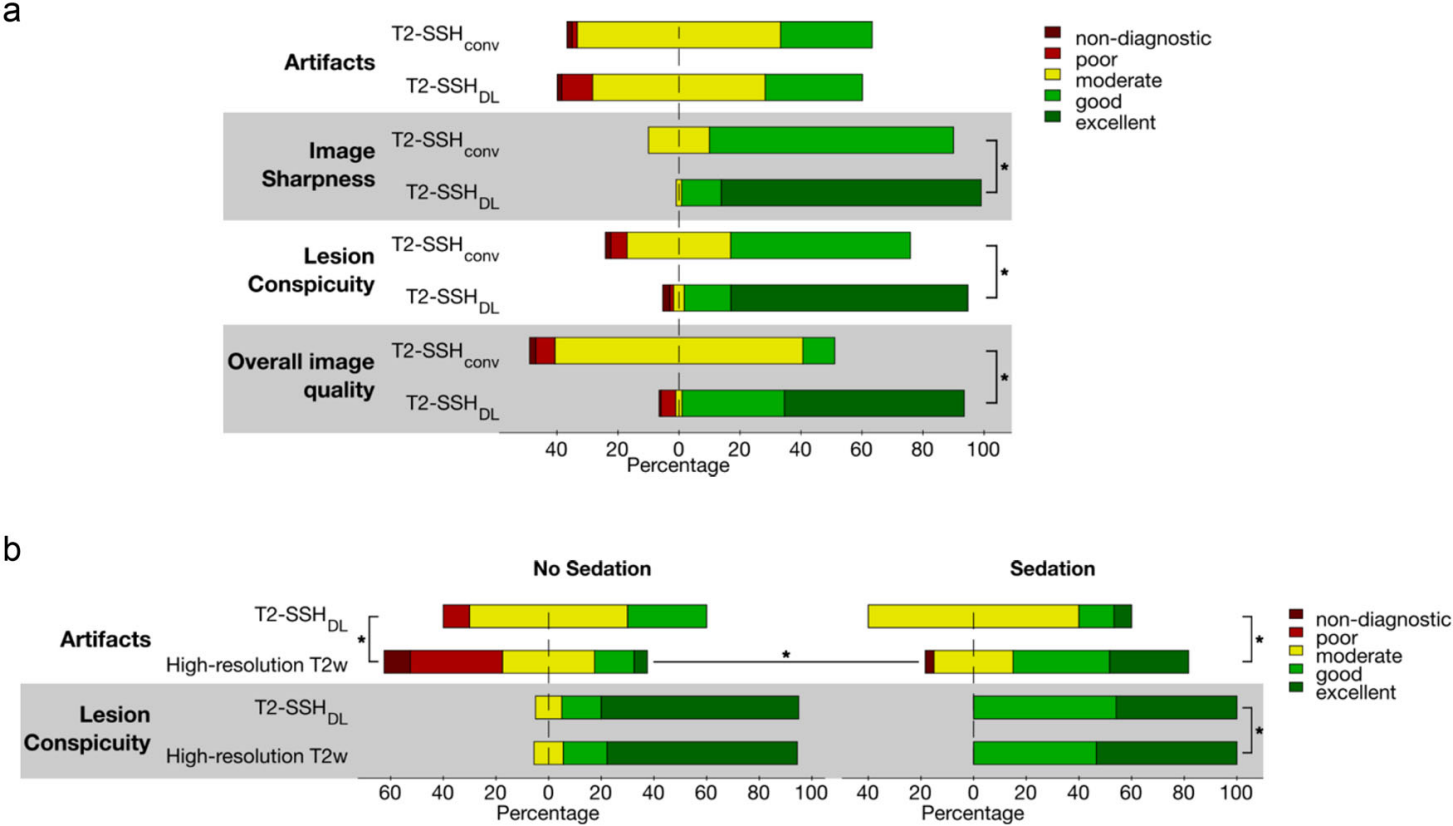
**a**, **b** Results of the qualitative assessment performed independently by two radiologists at time point 1. Stacked bar charts show the distribution of Likert scale scores stratified by sequence type. Statistically significant differences are indicated by an asterisk (*)

Interrater agreement was substantial, with Cohen’s κ of 0.71 (95% CI: 0.60–0.82) for the comparison between T2-SSH_conv_-sequences and T2-SSH_DL_-sequences, and 0.73 (95% CI: 0.53–0.93) for the comparison between T2-SSH_DL_-sequences and high-resolution T2-weighted sequences. Intrarater reliability was excellent with Cohen’s κ of 0.97 (95% CI: 0.93–1.00, rater 1) and 1.00 (95% CI: 0.99–1.00, rater 2) for the comparison between T2-SSH_conv_-sequences, and T2-SSH_DL_-sequences and 0.89 (95% CI: 0.75–1.00, rater 1) and 0.69 (95% CI: 0.47–0.91, rater 2) for the comparison between T2-SSH_DL_-sequences and high-resolution T2-weighted sequences. Table 3 and Fig. 4a, b provide the results of the qualitative rating performed by two radiologists, along with the *p*-values indicating the significance of the differences between the sequences.

**Table 3:** Qualitative evaluation per rater

		Artifacts			Image sharpness			Lesion conspicuity			Overall image quality		
Rater	Rating	T2-SSH_conv_	T2-SSH_DL_	*p*-value	T2-SSH_conv_	T2-SSH_DL_	*p*-value	T2-SSH_conv_	T2-SSH_DL_	*p*-value	T2-SSH_conv_	T2-SSH_DL_	*p*-value
1	1	4 (3–4)	4 (3–4)	0.008	4 (4–4)	5 (5–5)	< 0.001	4 (4–4)	5 (5–5)	< 0.001	4 (3–4)	5 (4–5)	< 0.001
	2	4 (3–4)	4 (3–4)	0.25	4 (4–4)	5 (5–5)	< 0.001	4 (4–4)	5 (5–5)	< 0.001	4 (3–4)	5 (4–5)	< 0.001
2	1	4 (3–4)	4 (3–4)	0.289	4 (4–4)	5 (5–5)	< 0.001	4 (3–5)	5 (5–5)	< 0.001	4 (4–4)	5 (4–5)	< 0.001
	2	4 (3–4)	4 (3–4)	0.289	4 (4–4)	5 (5–5)	< 0.001	4 (3–5)	5 (5–5)	< 0.001	4 (4–4)	5 (4–5)	< 0.001

Sedation							
		Artifacts			Lesion conspicuity		
Rater	Rating	T2-SSH_DL_	High-resolution T2w	*p*-value	T2-SSH_DL_	High-resolution T2w	*p*-value
1	1	4 (3–4)	4 (4–5)	< 0.001	5 (5–5)	5 (5–5)	0.07
	2	4 (3–4)	4 (4–5)	< 0.001	5 (4–5)	5 (5–5)	0.004
2	1	4 (3–4)	4 (4–5)	< 0.001	4.5 (4–5)	5 (4–5)	0.063
	2	4 (3–4)	4 (3–4)	< 0.001	4 (3–5)	5 (4–5)	< 0.001

No sedation							
		Artifacts			Lesion conspicuity		
Rater	Rating	T2-SSH_DL_	High-resolution T2w	*p*-value	T2-SSH_DL_	High-resolution T2w	*p*-value
1	1	4 (4–4)	4 (3–4)	0.002	5 (5–5)	5 (4–5)	0.25
	2	4 (4–4)	4 (3–4)	0.008	5 (4.75–5)	5 (4–5)	0.625
2	1	4 (3–4)	4 (3–4)	0.004	5 (4–5)	5 (4–5)	1
	2	4 (3–4)	4 (3–4)	0.38	4 (3.75–5)	5 (5–5)	0.002

Results of the qualitative evaluation comparing T2-SSH_conv_- and T2-SSH_DL_-sequences across all categories and raters. Median with interquartile range (IQR) and corresponding *p*-values indicate statistically significant improvements in image sharpness, lesion conspicuity, and overall quality for T2-SSH_DL_-sequences

**Fig. 5:**
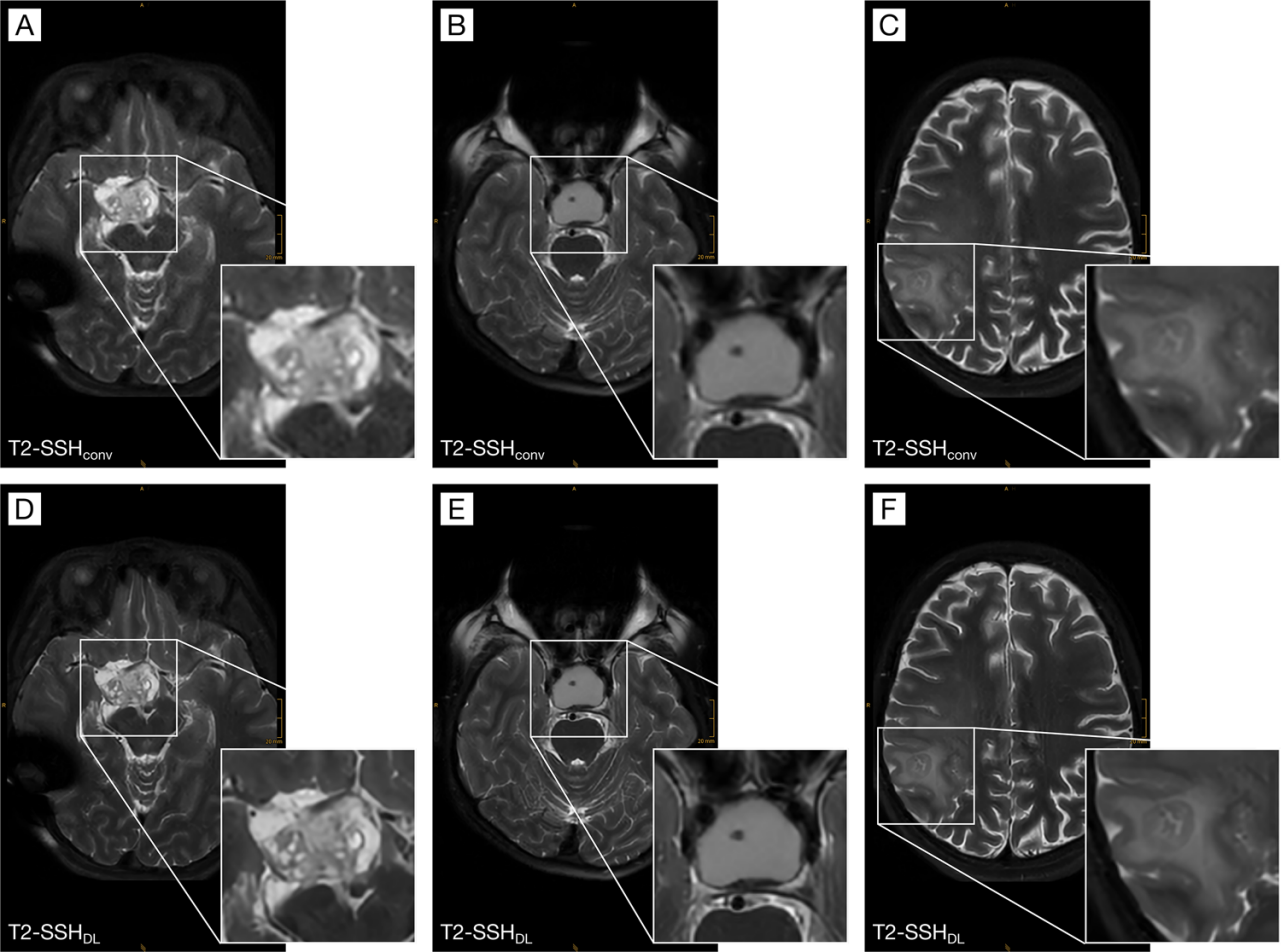
T2-SSH_conv_-sequence and T2-SSH_DL_-sequence in three pediatric patients. A 12-year-old boy with a suprasellar pilocytic astrocytoma (**A**, **D**), a 13-year-old boy with a craniopharyngioma after partial resection (**B**, **E**), and a 13-year-old boy with a glioma (**C**, **F**). Note the significant increase in image sharpness and excellent lesion conspicuity of the T2-SSH_DL_-sequence (**D**–**F**) compared with the T2-SSH_conv_-sequence (**A**–**C**) in all cases. A ventriculoperitoneal shunt on the patient’s right side in case **A** leads to imaging artifacts

**Fig. 6:**
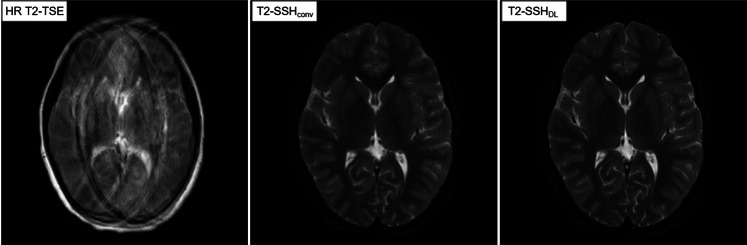
High-resolution (HR) T2-TSE sequence, T2-SSH_conv_-sequence and T2-SSH_DL_-sequence of an 8-year-old non-sedated boy with extensive motion artifacts. The HR T2-TSE sequence is prone to these motion artifacts, resulting in an image of poor diagnostic quality, whereas the single-shot sequences exhibit no motion corruption. Further, the T2-SSH_DL_ sequence shows markedly improved image quality

T2-SSH_DL_-sequences significantly increased overall image quality and showed improved image sharpness compared with T2-SSH_conv_-sequences. 33 of the 62 patients had lesions, including tumors such as gliomas, astrocytomas, craniopharyngiomas or embryonal tumors with multilayered rosettes; cystic lesions like pineal cysts, Rathke cleft cysts, choroidal fissure cysts or atypical Virchow space; focal areas of signal intensity (FASI) as a central nervous system manifestation of neurofibromatosis type 1; subcortical band heterotopia; post-treatment changes; and ventriculoperitoneal shunt placement. Overall, lesions were significantly more conspicuous in T2-SSH_DL_-sequences compared with T2-SSH_conv_-sequences, illustrated by representative cases in Fig. 5. The comparison of T2-SSH_DL_-sequences with high-resolution T2-weighted sequences showed that, in patients without sedation, T2-SSH_DL_-sequences provided fewer motion artifacts and comparable lesion conspicuity; thus, no lesions were missed. In sedated patients, high-resolution T2-weighted sequences achieved superior image quality and lesion conspicuity, as motion was minimized. Artifact analysis revealed a significant difference between sedated and non-sedated patients for high-resolution T2-weighted sequences, but no difference for T2-SSH_DL-_sequences, indicating reduced motion sensitivity. T2-SSH_DL_-sequences offered a significant advantage when strong motion-related artifacts were present in high-resolution T2-weighted sequences, as exemplified in Fig. 6.

For the comparison between T2-SSH_DL_-sequences and high-resolution T2-weighted sequences, only the categories artifacts, defined as the presence and severity of image artifacts including motion-related artifacts, blurring, ghosting, and ringing, and lesion conspicuity, were selected for evaluation. A direct comparison of overall image quality or image sharpness between these sequence types would not be appropriate, given their fundamentally different imaging characteristics. While high-resolution T2-weighted sequences inherently provide superior spatial resolution, single-shot sequences offer other advantages such as motion robustness and substantially shorter acquisition times. Therefore, this comparison primarily aimed to assess the effectiveness of motion-related artifact reduction by the single-shot technique and the ability to delineate lesions, for example, in follow-up imaging.

## Discussion

This study demonstrates that DL-based reconstruction can effectively overcome the fundamental trade-offs of accelerated MRI by improving both objective and subjective image quality without prolonging acquisition time. Compared with the conventional CS-reconstructed single-shot sequences (T2-SSH_conv_), the proposed DL-reconstructed single-shot sequences (T2-SSH_DL_) achieved significantly higher aSNR and aCNR and lower ERD, reflecting enhanced contrast, reduced noise, and improved spatial fidelity. However, despite statistically significant increases in aSNR and aCNR, these parameters exhibited considerable interindividual variability, likely reflecting acquisition- and patient-related factors such as coil configuration, sedation status, and slice thickness. Consequently, improvements in image sharpness represent a more robust and clinically relevant indicator of the benefits of DL-based reconstruction. Qualitative ratings confirmed these quantitative findings, showing significantly higher scores for image sharpness, lesion conspicuity, and overall diagnostic quality. Compared with standard high-resolution T2-weighted sequences, T2-SSH_DL_-sequences provided comparable lesion conspicuity and fewer motion artifacts in non-sedated children. Importantly, no lesion visible on standard T2-weighted sequences was missed on T2-SSH_DL_-sequences, indicating that diagnostic safety is preserved despite substantially shorter acquisition times. These improvements were consistent across orientations and independent of patient age or clinical indication, underscoring the robustness of DL-enhanced reconstruction in real-world pediatric settings [20]. Similarly, Ikebe et al confirmed in adults that dual-type DL-based reconstruction enables ultrafast whole-brain T2-weighted imaging within 7 s while maintaining diagnostic image quality, supporting the generalizability of DL-based acceleration across patient populations and imaging protocols [14].

It is important to emphasize that the observed motion robustness of the proposed approach and the feasibility of sedation-free imaging are primarily determined by the single-shot acquisition itself. The role of deep learning in this context is indirect: the DL-based reconstruction does not explicitly perform motion correction or suppression of motion-related artifacts, but rather mitigates image quality limitations inherent to single-shot imaging, such as noise amplification, signal decay–related blurring, and reduced spatial resolution. By improving denoising and spatial fidelity at ultra-short acquisition times, the DL reconstruction enhances the diagnostic usability of inherently motion-robust single-shot images, particularly in non-sedated pediatric patients.

These results align with previous reports showing that DL-based reconstruction can mitigate the inherent trade-offs of fast MRI by restoring spatial detail and contrast while maintaining short acquisition times [21]. DL-based reconstruction has been validated in adult MRI across multiple body regions, including the brain, prostate, musculoskeletal system, and heart, demonstrating superior sharpness, reduced artifacts, and higher diagnostic confidence under high acceleration factors [22–26]. Pediatric MRI provides an even more stringent benchmark for robustness and generalizability, as lower SNR, smaller anatomy, and frequent involuntary motion impose demanding imaging conditions. DL-enhanced single-shot imaging preserves lesion conspicuity and diagnostic confidence even in non-sedated children, outperforming conventional high-resolution T2 imaging under motion. This confirms that motion-robust single-shot acquisitions can remain diagnostically reliable where motion artifacts otherwise obscure pathology [27, 28].

Methodologically, integrating DL-based super-resolution into a CS pipeline addresses the intrinsic limitations of single-shot fast spin-echo imaging, signal decay and blurring along long echo trains [29, 30] without changing acquisition timing or hardware. This hybrid approach maintains the inherent motion robustness of single-shot acquisitions while significantly improving spatial detail and contrast. Comparable results have been demonstrated for DL-enhanced acceleration at different field strengths in cardiac cine imaging [31] and in musculoskeletal MRI [32], confirming the broad technological relevance of this reconstruction concept.

The clinical implications follow as a logical consequence of technological innovation. Diagnostic quality was maintained even in non-sedated children, indicating that motion-robust, DL-enhanced sequences can reduce or eliminate the need for sedation, a major clinical and logistical challenge in pediatric MRI [33, 34]. By producing high-quality, motion-robust images within seconds, DL-enhanced single-shot imaging could serve as a cornerstone of ultrafast, sedation-free pediatric brain protocols. This is particularly valuable for follow-up and shunt-surveillance examinations, where rapid yet reliable assessment is required. The established utility of single-shot MRI for hydrocephalus and other acute conditions provides a solid clinical foundation [35–37]. DL-based reconstruction extends this framework beyond narrow indications by mitigating the inherent constraints of spatial resolution and signal decay. The resulting improvements in workflow efficiency and diagnostic reliability are expected to enhance scanner throughput and reduce repeat imaging, emphasizing the importance of responsible, collaborative clinical implementation [38, 39].

While lesion detection proved reliable, lesion characterization remains superior on high-resolution T2 imaging, particularly for assessing internal cystic or fine structural components. Consequently, T2-SSH_DL_-sequences should be considered a complementary tool rather than a replacement for standard T2-weighted sequences. Its robustness supports consistent longitudinal imaging and may reduce the frequency of sedated examinations, thereby improving comparability across time points and enhancing patient safety.

Limitations include the use of vendor-specific reconstruction software, which may limit reproducibility across platforms, and the absence of pathology-specific diagnostic performance analysis. Importantly, the DL-based reconstruction does not explicitly model or correct patient motion, and its contribution to motion robustness should therefore be understood as indirect. Although image quality was assessed quantitatively and by expert consensus, future work should evaluate diagnostic accuracy for specific entities. The training data of the DL model may also influence performance, as previously observed in motion-corrected cardiac reconstructions and DL-enhanced musculoskeletal MRI [23, 32].

Prospective multicenter validation across field strengths, vendors, and age groups will be necessary before routine clinical implementation.

## Conclusion

This study demonstrates that a DL-based reconstruction framework can substantially enhance image sharpness, contrast, and diagnostic confidence while preserving short acquisition times. By integrating DL-driven super-resolution into a CS pipeline, the proposed approach effectively overcomes the intrinsic trade-offs between speed, noise, and spatial resolution that have long limited accelerated MRI. Validated in a motion-prone pediatric population, the reconstruction strategy improved the diagnostic usability of inherently motion-robust single-shot acquisitions under real-world, low-SNR conditions, maintaining lesion conspicuity and diagnostic reliability even in unsedated examinations. The combination of ultrafast acquisition and DL-enhanced reconstruction therefore provides a robust technical foundation for high-quality, clinically reliable, and potentially sedation-free pediatric MRI. Beyond its pediatric application, these findings underscore the broader potential of AI-driven image reconstruction to transform clinical MRI by translating algorithmic innovation into tangible diagnostic and workflow benefits.

## Abbreviations

aCNR: Apparent contrast-to-noise ratio
aSNR: Apparent signal-to-noise ratio
CI: Confidence interval
CNN: Convolutional neural network
CS: Compressed sensing
DL: Deep learning
ERD: Edge rise distance
GM: Gray matter
k-space: Spatial frequency domain of magnetic resonance imaging data
MC: Mastoid cells
MR: Magnetic resonance
MRI: Magnetic resonance imaging
PROPELLER: Periodically rotated overlapping parallel lines with enhanced reconstruction
ROI: Region of interest
SD: Standard deviation
SNR: Signal-to-noise ratio
T2-SSH_conv_: T2-weighted single-shot magnetic resonance imaging with conventional compressed sensing reconstruction
T2-SSH_DL_: T2-weighted single-shot magnetic resonance imaging with deep learning reconstruction
WM: White matter
